# Lady Hydrogen

**Published:** 1866-12

**Authors:** George Watt


DENTAL REGISTER.

Vol. XX.] DECEMBER, 1866. [No. 12.

### Original Communications.

### LADY HYDROGEN.

*Introductory to a course of lectures on Chemistry in the Ohio College of Dental Surgery.*

BY GEORGE WATT.

"Which of you, intending to build a tower, sitteth not down first, and counteth the cost?"

About attempting to build the superstructure of professional character on the foundation of professional attainments, may we not spend this hour in counting the cost? A high tower we propose to build; and great in proportion will be the cost. And he who is not willing to incur the necessary expense had best not attempt the superstructure; for only failure would result, "and all that behold it will begin to mock him, saying, this man began to build, and was not able to finish."

In view of the magnitude of the science to which I am expected to introduce you; in view of the great depths of ignorance into which we are all fallen; in view of the great heights to which we must climb to gain even a Pisgah view of the promised land of science; in view of the fact that my efforts to guide you will be far too much like the blind leading the blind; in view of the days and nights of mental toil and study in the months before us, it would be but human nature to spend this, the first hour of our acquaintance, in relaxation, if not in absolute rest. But shall we so spend it? Shall we not rather, with the aim of moving "immediately on the works," devote it to a review of our forces, and an inspection of the work that is before us?

But the scope of that science to which we are assigned is too vast for immediate consideration—too expansive for the survey of a single hour. It extends down to the foundations of the earth, and reaches up to Heaven. It includes all of earth, and air, and water, and meteors, and planets, and satellites, and comets, and stars, and suns. All material substances are subject to its laws; and the study of them is but the study of it.

Shall we, then, abandon the building of the intended tower? Having put our hands to the plow shall we look back? As soldiers marching against the battlements of ignorance, shall we desert our colors? No! but let us inure ourselves to the war by a gentle skirmish, before the grand battle begins. Let us spend this hour in the way of introducing ourselves to each other, and to our work. Like children "playing school," let us merely "make believe" that this is a lecture and you are students.

Then, of all the material universe, let us select, for present consideration, just one element. And, that our labor may not appear heavy, let us take that one which is the lightest and least tangible, the pliant, volatile, invisible, combustible little hydrogen.

Hydrogen! what inquiries crowd upon us with the very name! What is it? Where is it found? What is it like? What is it good for? and a score of others. Patience, my friends, let us consider.

Far away back in the long-ago, before "the morning-stars sang together," "or ever were formed the earth and the world," in the distant past, when "he spake and it was done," God said, "Let there be" and "there was" an element, which from the regal character bestowed on it by its Creator, not inaptly has been called "Lord Oxygen." And because it was not good for this lordly element to be alone, his Creator saw fit to provide "a help meet for him," and the beautiful hydrogen sprang up by his side, and their marriage was sealed by the lightning's flash, and announced by the thunder's roar; and, accordingly, we are told, in the family record of creation, that "darkness was upon the face of the *deep*, and the Spirit of God moved upon the face of the *waters*." Ye who worship at the shrine of antiquity, and bow in reverence to "first families," here is something worthy of your devotions. Here is the first recorded instance of definite chemical combination—the first marriage of elements. And so true have they been to each other, so close the tie of affection, so perfect the union, that for thousands of years, they were regarded by the whole human race, as "no more twain, but one" element.

What, then, is hydrogen? It is the lightest and most ethereal of all known substances. Too light to float the gossamer, or sustain the play of the zephyr's wing, too rare for the breath of the tiny insect, too diffused to carry the rose's perfume—a wonderful something which you can neither see, hear, feel, taste, or smell.

But where is it? Or, a simpler question, where is it not? It is above us, beneath us, around us, in us, of us. Go ask the forests the secrets of their growth, and they will tell you they have been watered and nurtured by the good Lady Hydrogen. Ask the flowers the source of their fragrance, and they will report it as a goodly present from the bride of Lord Oxygen. Admire their brilliant hues, and they will refer them to the artist hand of the same benefactress. Gaze at the rainbow, and you see but her pencilings. See her as she sparkles in the dewdrop, glitters in the rain, or glistens in the icicle. Admire her gentleness and purity, as she descends in the snowflake. Behold her majesty and grandeur, when the ocean is lashed to madness by the fury of the storm

Look at her stateliness in the floating iceberg, at her beauty in the shining frost crystal, at her tenderness in the falling tear. Explore the wildest, deepest, most remote recesses of nature, and you will find her in advance of you, beautifying, building up, and sustaining. Everything that lives is sustained by her; and deprived of her genial presence, would pine and die. And though practically almost omnipresent, yet she is never impertinent—never intrudes—neither meddles nor gossips. By her quiet presence, she manifests her willingness to serve those who need her sympathies. Though true to her first love, and faithful to her marriage vows, yet she willingly coöperates with the humblest member of the family of elements, if so she can be useful. Indeed, so modest and retiring is she that her presence is not suspected, even by those she blesses. "Many daughters have done virtuously," but she excels them all.

Such brilliance, and gentleness, and purity, and grandeur, and tenderness, and beauty, may well claim our attention and admiration for this hour. Shall we grow sentimental over the historic beauty of Helen, or ecstatic before the image of Venus, and not be filled with rapture in the presence of her who is unrivalled in beauty, and as pure as the angels, the beautiful bride of Lord Oxygen?

Would that I could but show you her portrait, but she shrinks from display. Like the timid maiden of the East, she refuses to unveil. Like the modest wife, she is content that "her husband is known in the gates, when he sitteth among the" elements. She is like the silent benefactor—"she stretcheth out her hands to the poor, yea, she reacheth forth her hands to the needy." She is like purity itself. The touch of filth does not defile her; nor can the breath of slander defame her. And though her beauty and loveliness may sometimes prove her misfortune, and those chemical bandits, sulphur, chlorine, and phosphorus, may kidnap and carry her off, they find a constancy they can not overcome, charms not for them; and, instead of the pleasures they expected to enjoy in her constrained presence, all is acrid and sour; and when her lordly husband comes to her rescue, and consumes these robbers with the breath of his mouth, spotless and undefiled, his own well beloved flies to his embrace, and "the heart of her husband doth safely trust in her."

But the gentleness, brilliance, stateliness, grandeur, and beauty of this wonderful lady element are all as nothing when compared with her goodness. While others bestow favors for applause, she blesses for the sake of blessing. So gentle, airy, and light, we would expect her to play with the gossamer, or romp with the butterfly, or to soar away up above the clouds, and sport in the blue ether, among the stars and comets. But had she the strength of iron, the gravity of lead, and the caustic energy of chlorine combined, she could not undertake or accomplish more than she does. Her power is wonderful, and, like the true lady, she rules only by love; and so gentle is her sway that her subjects are her willing slaves, and are all unconscious of their servitude. And even her lordly husband, with all his energies and passions, is overcome by her affection, and in her embraces he is as gentle as a lamb. Alone, the leaves of the forest wither in the blast of his breath, and nature is defaced by his blighting touch. Soothed by her smiles, he waters the earth, and covers it with its carpet of grass, while the forest is clothed with leaves and flowers, and all nature puts on her holiday garb. Alone, he burns cities to ashes, or crumbles them to dust. Captivated by her gentle sway, he toils like a slave to extinguish the conflagration. Alone, he breaks our ships with his east wind. With her influence and aid, he bears them up, and floats them to the desired havens. Alone, he forges the thunderbolts of war, and hurls them forth on their errands of death. With her, he quenches the thirst of the wounded and dying, and cools the parched lips and fevered brows of the hospitals' inmates. Alone, he devours the widow's bread, and destroys the fruits of the ground to create the demon-drink that fills the world with crime, and changes men to devils. Blest with her genial presence, he furnishes the clear, cool water, which entails only blessings on those who drink it. In many of his grandest and best achievements, he would be utterly powerless, without her presence and her aid. Let us think of this.

"In the day that God created" these wonderful elements, positive and negative "created He them, and blessed them and called their name WATER, in the day when they were created." Life-sustaining, health giving water! The drink that a God of love has distilled for all his children! The liquor that strengthens, refreshes, revives, invigorates, and purifies. The beverage which has no orphan's tear as its sequel, nor widow's wail as a requiem over its fallen victim. A drink that demons do not delight in. The murderer does not imbibe it to prepare him for his crime, nor the reveller as a prelude to his midnight debauch. No ghosts of murdered innocents awake from their slumbers to curse the cup that contains it, or the fountain that gushes it forth. Dethroned reason does not curse and babble under its influence; nor does delirium travel in its wake. The lone prisoner does not accuse it of the crime that has brought him to the dungeon-cell; nor does the felon on the scaffold, curse "the moss-covered bucket" for his untimely end. Prisons and almshouses do not overflow with its victims; nor are courts of justice kept busy with its crimes.

No! our Lady Hydrogen and her liege lord, are, in combined labors of love, far otherwise engaged. Like their divine Creator, they go about doing good. They are his agents in preparing rain, and making grass to grow upon the mountains. By them he visits the earth and waters it, and enriches it with the river of God. They are the messengers by which he sends the mollifying shower and the refreshing dew. Out of them is formed the storm-cloud and the rivers, and the seas and the ocean. Nor are they easily baffled in their benign efforts. They impart power to the majestic steamer, and it stems the river's current, and rides the angry billows.

Nor is any work too servile for them, if only it be useful. They turn the mill-wheel and the spindle, and draw the produce of the farmer to the distant market, and return laden with the wares of merchandise. They float the mariner's stately ship, and the red man's bark canoe. They wash the poisonous malaria from the atmosphere, and the filth from the surface of the earth, to impart health and prolong life. They never weary in well doing, but sustain and bless everything that lives, and without their presence, life is impossible.

And with all their usefulness, they do not forget to be ornamental, nor, while supplying necessities, do they ignore luxuries. They humor all our tastes, and gratify all our senses. They cool the fevered brow, moisten the parched lips, and quench our burning thirst. And how they charm our sight. By the north wind's aid, they show us the hoar-frost, the ice-crystal, and the snow-flake. By the help of the vernal breezes, they paint the flowers, bedeck the forest, and cover the earth with its carpet of green. They hold up the storm-cloud as a back-ground to the rainbow of promise. They furnish "a way for the lightning of thunder," and thus display to us its grandeur. They hang up, as a curtain, the fleecy cloud, and adorn the sunset with its crimson veil. They take up and bear to us the fragrance of the flowers, as sweet as the perfumes of Araby the blest. And who has not listened with rapture to their music?

"What a joy to press the pillow
Of a cottage chamber bed,
And to listen to the patter
Of the soft rain overhead!"

Who has heard, elsewhere, such music

"As that melody of nature—
That subdued, subduing strain,
Which is played upon the shingles,
By the patter of the rain?"

And the purling brook, and the warbling rill—whose childhood has not been delighted with their melodies? And the merry cascade

> " * * as it gushes,
> Like a flow of laughter, out—"

Who has not felt the power, but who can describe the sweetness of its strain?"

Thus does our Lady Hydrogen, like a true and faithfu wife, coöperate with her lordly husband. His glory is hers and her proudest thought is of her relationship to him. He is king among the elements, she his early-loved, and acknowledged queen.

But, though her delight is to assist and coöperate with him; yet when duty calls him from her side, she is neither despondent nor idle. If he kindles a fire for our comfort, she contributes to the fuel. Just think of her going down into the dark caverns of the earth ages ago, and there embracing her dark-eyed brother carbon, that she might bring him up, at the proper time, to furnish us the heat and light whose comfort and cheer enable us to spend this hour together, and, thus thinking, you can appreciate a little of her self-denial. And, though not able to rule the other elements, like her royal husband, yet she often accomplishes as much by management as he does by force. Is that refractory element, gold to be subdued? she coaxes the bandit chlorine into her service, guides him to the culprit, and, during the contest that ensues, returns to the side of her lord. Does chlorine refuse to surrender the captive to the demand of lawful authority? She forms an alliance with sulphur, another of the banditti, who, over-coming his late comrade, carries off the prisoner, but abandons him to freedom, through fright at the heated anger of Lord Oxygen. Is the benighted and bewildered traveller to be warned against fatal bogs and morasses which lie in his pathway? she plunges into the mire in search of her fiery brother Phosphorus, and, with loving embrace, lures him to the surface, and, in the light of a beacon flash rejoins her Lord.

But what shall I say more? The time would fail me to tell of her marvellous works, and her benificent deeds. She is a wonderful work of a more wonderful Creator. If this is but a passing glance at one of the elements he has made, how extensive the study of them all! If this is the lightest and rarest of all, yet possessed of properties wider than the range of our thoughts, and beyond the reach of our comprehension, how vast the field of science which includes the properties of all of them!

Are you, then, discouraged at the prospect? Do your hearts fail you? Take courage, my friends! The voyage of the world is a long one—possibly too long for your strength and means—but is that any reason you should not enjoy a pleasure-walk? There are foreign countries you would like to see. Deprived of visiting them, must you fail to call on your friends in the next village? The greatest charm of the science we propose to study, is its almost infinite extent. It will last us for life; and I hope it will last us for eternity, and that we will all study it in the same class. But, while it is not possible for you to get through with the study of chemistry in your college course which now commences, it is quite practicable for you to acquire such a knowledge of its principles as will make all future study of it a pleasure rather than a toil, and such as will enable you to apply many of these principles to the wants of the profession to which you aspire.

In the months that we are to be together, it will not only be my official duty, but also my special pleasure, to aid you in the study of this science. It is regarded as a dry subject, but you will not find it so, if you endeavor to understand it. In the name of the faculty, I welcome you to our halls.

